# Low Message Sensation Health Promotion Videos Are Better Remembered and Activate Areas of the Brain Associated with Memory Encoding

**DOI:** 10.1371/journal.pone.0113256

**Published:** 2014-11-19

**Authors:** David Seelig, An-Li Wang, Kanchana Jaganathan, James W. Loughead, Shira J. Blady, Anna Rose Childress, Daniel Romer, Daniel D. Langleben

**Affiliations:** 1 Annenberg Public Policy Center, Annenberg School for Communication, University of Pennsylvania, Philadelphia, Pennsylvania, 19104, United States of America; 2 Department of Psychiatry, School of Medicine, University of Pennsylvania, Philadelphia, Pennsylvania, 19104, United States of America; Leibniz Institute for Neurobiology, Germany

## Abstract

Greater sensory stimulation in advertising has been postulated to facilitate attention and persuasion. For this reason, video ads promoting health behaviors are often designed to be high in “message sensation value” (MSV), a standardized measure of sensory intensity of the audiovisual and content features of an ad. However, our previous functional Magnetic Resonance Imaging (fMRI) study showed that low MSV ads were better remembered and produced more prefrontal and temporal and less occipital cortex activation, suggesting that high MSV may divert cognitive resources from processing ad content. The present study aimed to determine whether these findings from anti-smoking ads generalize to other public health topics, such as safe sex. Thirty-nine healthy adults viewed high- and low MSV ads promoting safer sex through condom use, during an fMRI session. Recognition memory of the ads was tested immediately and 3 weeks after the session. We found that low MSV condom ads were better remembered than the high MSV ads at both time points and replicated the fMRI patterns previously reported for the anti-smoking ads. Occipital and superior temporal activation was negatively related to the attitudes favoring condom use (see Condom Attitudes Scale, Methods and Materials section). Psychophysiological interaction (PPI) analysis of the relation between occipital and fronto-temporal (middle temporal and inferior frontal gyri) cortices revealed weaker negative interactions between occipital and fronto-temporal cortices during viewing of the low MSV that high MSV ads. These findings confirm that the low MSV video health messages are better remembered than the high MSV messages and that this effect generalizes across public health domains. The greater engagement of the prefrontal and fronto-temporal cortices by low MSV ads and the greater occipital activation by high MSV ads suggest that that the “attention-grabbing” high MSV format could impede the learning and retention of public health messages.

## Introduction

Health-related video public service announcements (PSAs) and commercial advertisements promoting health-related products and healthy behaviors, are an important component of public health campaigns to reduce the incidence of sexually transmitted infections (STI) and addictions [1], [2], [3]. To facilitate objective evaluation of health-related video advertisements (further “ads”), researchers have developed several standardized measures of video ad characteristics. “Message sensation value” (MSV), which quantifies video ad features that are arousing and engage attention, is one of the best validated of such measures [4], [5], [6], [7]. Data support MSV’s construct validity and value in predicting behavioral outcomes of video ads [8], [9], [10]. While there is no objective algorithm for development of effective video ads, a long held consensus has been that high MSV is likely to increase video ad effectiveness by capturing more attention [4]. This consensus was also in-line with the “distraction hypothesis” stating that diversion of attention from an ad core message to its other features (i.e., distraction), may actually *enhance* message effectiveness by impeding a viewer’s ability to argue against an ad’s arguments [11]. An alternative point of view was that attention-grabbing audio-visual features that confer higher salience may actually compete with ad’s content for limited cognitive resources, thus reducing the processing of an ad’s message [6], [12], [13], [14], [15], [16]. In line with the latter overstimulation interpretation, a functional Magnetic Resonance Imaging (fMRI) study of anti-smoking video ads [17] found that compared to high MSV ads, low MSV ads were associated with better recognition of still frames extracted from these ads after a short delay and were more likely to activate the middle temporal gyrus during viewing, one of the brain regions associated with memory formation (i.e., encoding) [18], [19]. In addition, low MSV ads were more likely to activate areas of the prefrontal cortex associated with top-down attention and cognitive processing [20], [21], [22]. In contrast, high MSV ads were associated with greater activation of the occipital cortex, a region associated with initial processing of visual information [23], [24].

The above findings suggest that high MSV ads may interfere with memory encoding, perhaps due to the increased allocation of the limited cognitive resources to visual processing. Consistent with this hypothesis, medial temporal and prefrontal response to low MSV anti-tobacco video ads was positively correlated with subsequent frame recognition accuracy [17].

Despite the evidence that high MSV may disrupt the processing of ad arguments, campaign designers often include high MSV inputs to capture attention and without considering possible negative effects of these distractors [25]. The present research was designed to provide a further test of the “limited cognitive resources” explanation for the poorer recognition accuracy of high MSV ads. In particular, we investigated the relationship between regions associated with sensory stimulation during ad viewing (primarily occipital cortex) and the prefrontal and temporal regions associated with cognitive processing and memory encoding. If the “limited cognitive resources” explanation is valid, we would expect the connectivity between these regions to be weaker under high MSV than low MSV. The presence of such a system is supported by anatomical evidence of a direct occipito-temporal network [26], as well as functional demonstrations of an association between these regions during encoding of sensory information [27], [28], [29].

A second goal of the research was to determine whether the superiority of low MSV ads previously demonstrated for anti-tobacco video ads [17] would extend to another important public health domain, namely safe sex. We anticipated that prior findings of superior encoding of low MSV ads would generalize to safe sex video ads promoting condom use. Generalization of the findings from anti-tobacco video ads to an entirely different topic would replicate the prior finding and suggest that it is relevant to a wider range of health topics. We hypothesized that low MSV ads will be recognized on subsequent tests of recognition memory better than high MSV ads. Furthermore we hypothesized that low MSV will preferentially activate temporal and prefrontal cortices, while high MSV ads would preferentially activate the occipital cortex. To explore our hypothesis that low MSV ads are associated with a greater degree of occipito-temporal connectivity than high MSV ads, we examined the psychophysiological interaction (PPI) between the occipital and the prefronto-temporal regions. Such a relationship would suggest that video ads with excessive visual stimulation prevent the efficient transfer of information from regions involved in visual processing to downstream regions associated with storage of this information in memory.

## Materials and Methods

### Participants

Thirty-nine healthy subjects were recruited through advertising and screened for psychiatric, neurological or medical illnesses. Exclusion criteria included presence of DSM-4-TR Axis 1 psychiatric disorder [30], chronic medical or neurological illness or treatment that could alter cerebral structure or metabolism, and safety-related contraindications for MRI scanning. All participants reported sexual experience. Data from 34 subjects (17 F), aged from 19 to 31 years (Mean 23.4, SD 2.9), with an average of 15.2 (SD 1.7) years of education, all right handed, were included in the final analysis. Among these participants, 11 (33%) self-identified as African American, 16 (47%) as European American and 7 (21%) as Asian American. All participants reported having been sexually active in the past three months with an average of 1.4 (SD 0.7) sexual partners. Six (2 M) reported having sexual partners of both genders and 10 (4 M) reported never using condoms. Each participant gave written informed consent to participate in the study. The study protocol and consent form were approved by the University of Pennsylvania Institutional Review Board.

### Stimulus selection

We identified 71 ads that encouraged condom use by populations at-risk for unplanned pregnancy or sexually transmitted Diseases (STDs). Most of the ads were commercials produced by condom manufacturers (e.g., Trojan, Durex), who share a common goal with government-sponsored programs to encourage their target audiences to engage in safe-sex practices. Any references to particular manufacturer brand names were cut or digitally blurred. All 71 videos were scored for MSV by two raters who were trained on using the validated rating procedure [31], [32]. Briefly, MSV is an aggregate measure of audio and visual format features of video messages, derived from Zuckerman’s theory of sensation-seeking [33]. The rated features include cuts, special effects, intense graphic imagery, saturated colors, loud sounds, animation and so on. The specific MSV rating procedure used in this study has been described in Morgan et al [31] and validated in multiple previous studies [15], [34], including studies with neurophysiological correlates [17], [32], [35]. In the present study, the MSV of each ad was coded independently by two raters following the procedure described by Morgan et al. (2003). Rating are based on features described above, adjusted for ad length [31]. An ads’ total score is a composite of both qualitative (Present/Absent) and categorical (number of occurrences) ratings. The inter-rater correlation for the total score ratings in the present set of ads was very high (Pearson r = 0.96). The rare divergent ratings were reconciled by consensus between the two raters and a 3^rd^ similarly trained individual.

We selected 32 complete ads (ranging between 26 to 66 [x¯ = 42.2] seconds in length) from the set of 71 drawn equally from above and below the collection’s median MSV score of 6 (mean 6.2, SD = 2.9). The length of the ads was not different between high and low MSV categories (high MSV: 40.81±12.06; low MSV: 40.75±13.64; t = 0.01, df = 30, p = 0.99). We created two sets of 16 ads, each consisting of 8 “high MSV” and 8 “low MSV” ads, with no difference in MSV between these two sets (t = 0.30, df = 30, p = 0.68). The mean MSV of the low subgroup was 3.6 (SD = 1.2, range = 1–5), and the mean MSV of the high subgroup was 8.8 (SD = 1.3, range = 7–10). MSV values were significantly different between low and high MSV ads (t = −11.3, df = 30, p<0.001). Four of the selected videos were produced by firms that sell condoms (e.g., Trojan, Durex), while the rest were developed by non-profit or government public health agencies (e.g., Centers for Disease Control) for network and cable broadcast (e.g. MTV).

Wang et al. [35] found an interaction between MSV and the persuasive strength of an ad’s argument (AS). As the goal of the current study was to compare the effects specifically associated with variation in MSV, we assessed whether AS was balanced between the two MSV conditions, using a previously employed questionnaire [32], [36] immediately prior to the end of their participation in the first session of the study. The mean AS scores of high MSV ads did not significantly differ from the mean AS of low MSV ads (t = −0.007, df = 30, p = 0.994).

### Video task

**Figure 1:**
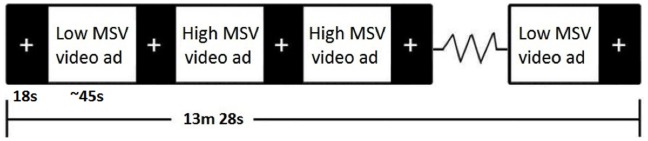
Design of the video message task. The actual task displays 16 video messages (8 high MSV and 8 low MSV) in pseudorandom order (the order presented above is one possible organization).

Subjects were given the task (see Figure 1) of viewing one of the sets of 16 videos presented in a random sequence. Each ad was preceded and followed by an 18-second interstimulus interval (ISI) during which a homogenous black background with a grey fixation point (“+”) was displayed. These ISI’s were used as baseline periods for the purpose of imaging data analysis. Stimuli were not repeated and total task duration was 13 min 28 s.

### Frame Recognition Test

Five minutes after the video task, subjects were given the Frame Recognition Test (FRT), a forced-choice recognition memory task designed to probe episodic memory of the videos they had seen in the scanner. This task was adapted from an electroencephalography study of the retention of TV commercials [37]. The FRT used in the current study consisted of 3 still-frame targets extracted from each of the 16 videos seen by the subject and 3 still-frame foils from each of 16 videos not seen by the subject (96 frames in total). To construct the frame recognition task (FRT) we used the approach of Wang (2013), Langleben (2009), and Silberstein (2001). Briefly, six frames, sampled at equal time intervals from each other to ensure that they were representative of the full length of the ad, were extracted from each ad. Then we randomly chose 3 frames from each video for the initial and follow up recognition tests. Frame stimuli were presented for 3 second each, in an optimized pseudo-random order [38] that included a variable ISI (0.25 to 16.25 s) during which a fixation point was present (see previous section above). For each trial, participants were instructed to respond “Yes” or “No” to the question “Have you seen this ad?”, using a two-button keypad (FORP, Current Design Inc., Philadelphia, PA). The question intentionally implied the ad as a whole though only a single frame was displayed. Stimuli were not repeated, and the total task run time was 9 min 22 s.

### Delayed Frame Recognition Test

Three weeks following the fMRI session, participants returned for a second Frame Recognition Test to examine decay and to assess whether initial differences between high MSV and low MSV ads remained. The FRT presented during this session (“delayed FRT”) used the same design as the FRT that immediately followed the ad-viewing task. However, each participant was presented with a different set of 96 still-frames (also extracted from the same viewed and unviewed videos as the first FRT).

### Condom Attitude Scale (CAS)

Subjects were questioned about their attitudes towards condoms immediately before and after viewing the video task using an abridged version of the Condom Attitude Scale (CAS) [39]. The abridged CAS contains 17 items that query three factors: condom efficacy in preventing sexually transmitted diseases (STDs) and perceived risks of STD, interpersonal impact of condom use, and the effect of condoms on sexual experience. Participants were instructed to indicate the extent to which they agree or disagree with each statement on a 1–6 scale (1 = Strongly Disagree, 6 = Strongly Agree). Two-way repeated-measures ANOVA assessed the main effect, on CAS, of the within-subjects variable (before vs. after task) and the between-subject variable (male vs. female), as well as their possible interaction.

### Procedure

All visual tasks were programmed in the Presentation (Neurobehavioral Systems Inc., Albany, CA) stimulus presentation package and rear-projected to the center of the visual field using a PowerLite 7300 Video projector system (Epson America, Inc., Long Beach, CA) that was viewed through a mirror mounted on the scanner head coil. The video soundtrack was delivered through Silent Scan 2100 MRI-compatible headphones (Avotec Inc., Stuart, FL). Before the imaging session, participants were instructed to watch all the videos carefully, that their memory of the videos would be tested after a delay and that their performance on this test was an integral part of the experiment. Participants performed the FRT approximately five minutes after the video task. The FRT was repeated 3 weeks later at a second session.

### Behavioral data analysis

Statistical analyses were performed using the IBM Statistical Package for the Social Sciences (IBM SPSS version 21). Subjects’ performance on the FRT was evaluated using the Discrimination Index, Pr = Z_Correct target recognition_–Z_False alarms_, which reflects how well subjects correctly distinguish targets from foils [40]. All subjects performed above chance level (Pr≥0), so none were excluded from analyses. Subject tendency to respond “Yes” or “No” under uncertainty was evaluated using the Response Bias measure, Br = Z_Correct target recognition_+Z_False alarms_. Br = 0 indicates no bias, Br<0 indicates liberal bias (i.e. tendency of saying “Yes” when uncertain), Br>0 indicates conservative bias (i.e. tendency of saying “No” when uncertain). Paired t-tests were performed comparing subject performance on Session 1 vs. Session 2 FRT (immediate vs. delayed sessions), and high MSV vs. low MSV. Independent t-tests were performed comparing gender performance.

### fMRI data acquisition

MRI data were collected on the University of Pennsylvania’s Siemens Trio (Erlangen, Germany) 3 Tesla whole body system using a 32-channel head coil. BOLD fMRI gradient-echo echo-planar sequence was acquired with the following parameters: TR = 3000 ms, TE = 32 ms, flip angle = 90, matrix = 64×64, FOV = 220 mm × 220 mm, slice thickness/gap = 3.4/0 mm, 46 slices. After BOLD fMRI, a 5-min magnetization prepared, rapid acquisition gradient echo T1-weighted image (MPRAGE, TR = 1620 ms, TE = 3.87 ms, FOV 250 mm, Matrix 192×256, effective voxel resolution of 1×1×1 mm) was acquired covering the whole brain for spatial normalization and anatomical overlay of functional data [41].

### fMRI data preprocessing

fMRI data were preprocessed and analyzed using FEAT(fMRI Expert Analysis Tool), part of FSL (FMRIB’s Software Library, www.fmrib.ox.ac.uk/fsl). Images were slice time-corrected, motion-corrected to the median image using tri-linear interpolation with six degrees of freedom [42], high-pass filtered (100 s), spatially smoothed (5 mm FWHM, isotropic) and scaled using mean-based intensity normalization. The median functional and anatomical volumes were co-registered and then transformed into standard space (T1 MNI template) using tri-linear interpolation [42], [43]. BET (Brain Extraction Tool) [44] was used to remove non-brain areas. Data sets with motion or signal-to-noise ratio exceeding 2 standard deviations from the average were excluded (5 of 39 initial subjects). Coordinates were converted to Talairach space [45] for tables and figures.

### Subject-level Analysis

Subject-level time series statistical analysis was carried out using FILM (FMRIB’s Improved Linear Model) with local autocorrelation correction [46]. High MSV and low MSV video messages were modeled using a canonical hemodynamic response function for all subjects based on the order of stimulus presentation for each subject. Six rigid body movement parameters from the motion correction were modeled as nuisance variables. Second level analysis was performed on the contrast images generated from the subject level analysis including: high MSV>low MSV; low MSV>high MSV; All videos>baseline. Age, gender, educational level and condom use status were entered as covariates of no interest. Group statistical maps were thresholded at z = 2.3. To control for Type 1 error, group maps were cluster corrected for multiple comparisons at p<0.05, using the Family-wise Error Rate based on Gaussian Random Fields (GRF) theory. Brain response to high and low MSV ads was compared between African American and Caucasian participants. Lastly, we analyzed the correlation between brain activation during ad viewing and the change in CAS after the fMRI session, calculated as ΔCAS = CAS_post – CAS_pre, which was entered as a covariate of interest for the All videos>baseline contrast, resulting in positive and negative correlation maps (z>2.3 p<0.05).

### Psychophysiological Interaction (PPI) Analysis

**Figure 2:**
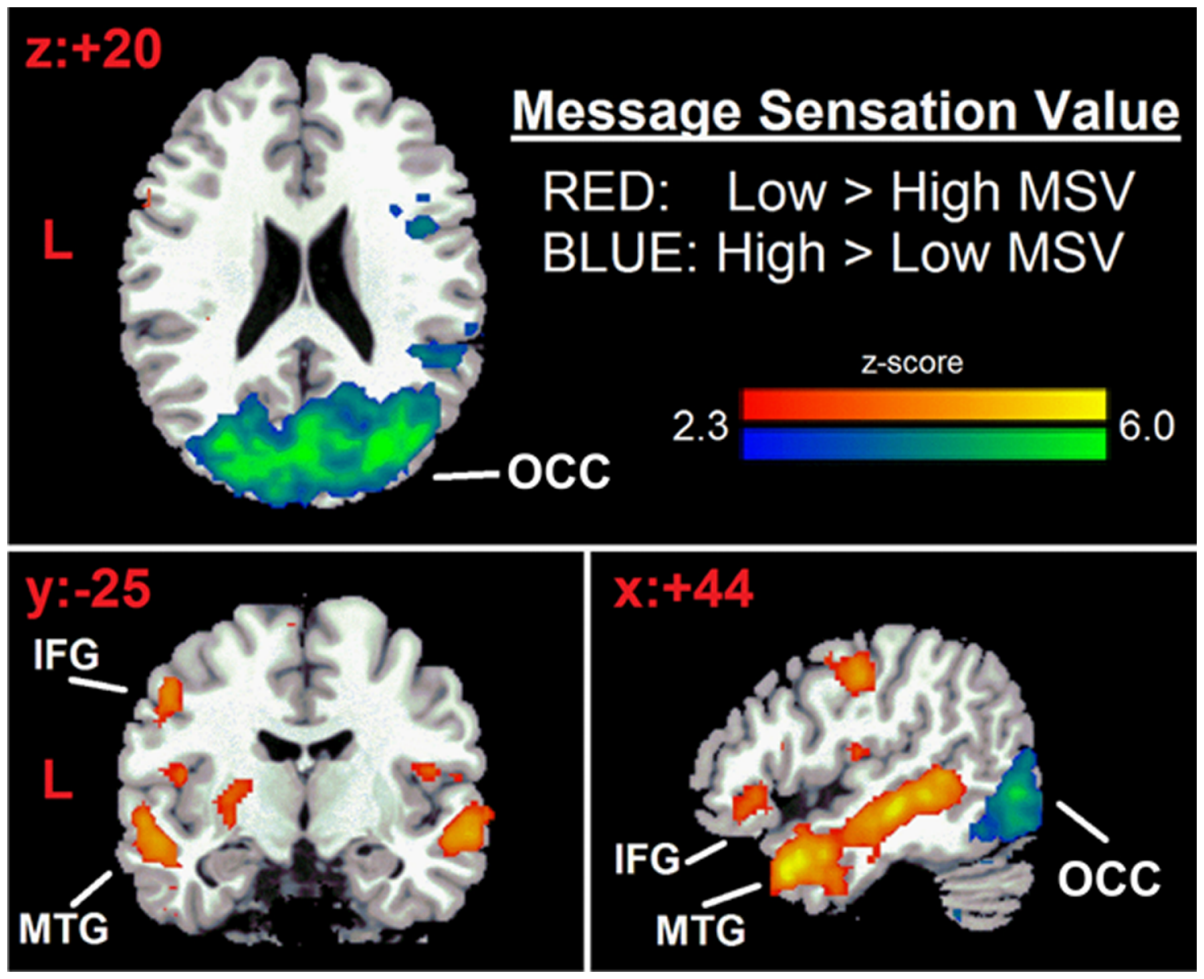
Brain response to safe-sex video messages. Middle temporal gyrus (MTG) and inferior frontal gyri (IFG) (red) have increased response for Low MSV>High MSV items. Occipital cortex (OCC) (blue) has increased response for High MSV>Low MSV ads. Statistical maps are displayed over the Montreal Neurological Institute (MNI) brain template and thresholded at Z = 2.3, cluster-corrected for multiple comparisons at p<0.05. Coordinates converted to Talairach space [45].

The PPI analysis was designed to test the hypothesis that greater visual processing would interfere with memory encoding of the ads. Thus, brain regions most associated with visual stimulation (specified by the **high** MSV>**low** MSV contrast) would have a weaker or negative functional relationship with the brain regions required for successful memory encoding of the videos, specified by the low MSV>high MSV contrast in the High MSV condition than the low MSV condition. In the first step of this analysis, based on methods detailed by Gitelman et al. [47], we computed individual average time-series to modeled interactions (for the two MSV conditions separately) between each voxel in the brain and a seed region specified by a functional bilateral mask. This mask was created by using the voxels surviving cluster correction (z>2.3 p<0.05) in the high MSV>low MSV contrast. As seen by the blue-tinted voxels in the left half of Fig. 2, this seed mask was also nearly identical to the anatomically-defined occipital cortex. The seed time-courses for each subject were deconvolved based on the canonical hemodynamic response function (HRF) used by Gitelman et al [47] to construct a time series of neural activity in this region for each MSV condition. We then estimated a GLM with three regressors specifically during video-viewing segments: (1) A binary indicator function specifying low MSV vs. high MSV trials, (2) Average BOLD time-series for the seed (OCC mask) described above, and (3) An interaction between neural activity in the OCC seed and the indicator function described in #1. The second and third regressors were convolved with the HRF.

As our hypothesis was restricted to interactions between regional networks functionally defined by our task (i.e., brain areas associated with sensory stimulation vs. those associated with strong memory encoding), we utilized a second functional bilateral mask as a target region of interest (ROI). This seed mask was created by using the voxels surviving cluster correction (z>2.3 p<0.05) in the low MSV>high MSV contrast. As seen by the red-tinted voxels in Fig. 2, this mask primarily covers the medial temporal gyrus (MTG) with a smaller proportion of voxels covering the inferior frontal gyrus (IFG). Our subsequent analysis examined the magnitude and valence (positive or negative) of each voxel in the ROI target based on its interaction with the seed during our task (i.e., we did not investigate any seed-voxel interactions outside of the target ROI mask).

To compare the strength and direction of averaged interactions involving the OCC (seed) and the MTG/IFG (ROI target) between low MSV vs. high MSV conditions, we performed a second-level t-test analysis using a procedure modified from Hartley et al [48]. As each subject (N = 34) possessed a pair of average OCC – MTG/IFG interaction values for the low MSV and high MSV conditions respectively, we calculated a two-tailed paired t-test to look for significant interaction differences between the two conditions.

To further characterize differences between the two PPI distributions, we divided both conditions’ voxel distributions into two sets of 5 quintiles and used paired t-tests to compare the means within each pair of corresponding quintiles. This analysis involved the following procedure: Using the OCC mask as the seed region, a PPI z-score was determined for each voxel within the MTG/IFG (target region of interest, ROI) for each subject. Within the target ROI, the voxel/seed PPI distributions were broken into two sets of quintiles, one set for each condition.

The goal of this analysis was to determine whether differences in interaction between low MSV versus high MSV were driven by (1) a relative increase in positively interacting voxels during low MSV, (2) a relative increase in negatively interacting voxels during high MSV, or (3) a combination of these mechanisms. We also sought to determine if low MSV’s more positive PPI mean could be associated with a weaker overall associative relationship between the seed and target ROI relative to high MSV, a result which would disprove our hypothesis that low MSV videos improve the cooperation between regions involved in input of sensory information and downstream encoding of this information.

## Results

### Behavioral data

A two-way repeated ANOVA revealed that memory performance was significantly better during the immediate post-task FRT session than the 3-week delayed session (F = 101.13, df = 1, p<0.001), and low MSV ads were bettered remembered than high MSV ads (F = 60.68, df = 1, p<0.001), but no interaction between Session and MSV (F = 2.20, df = 1, p = 0.15). Subjects responded significantly less conservatively during low MSV than high MSV ads (Br = −0.099 vs. 0.24, t = −7.045, df = 33, p<0.001). There were no significant bias differences between sessions (Br = 0.15 vs. 0.69, t = 1.36, df = 33, p = 0.183). An independent t-test on gender revealed no significant differences between males vs. females in discrimination or response bias (Pr = 2.05 vs 2.20, t = −0.775, df = 32, p = 0.444; Br = 0.033 vs. 0.18, t = −1.67, df = 32, p = 0.104). Examining the pre- and post-task CAS measure, there was no main effect of PSA exposure (F = 0.546, df = 1, p = 0.465) or interaction of task and gender (F = 0.076, df = 1, p = 0.784). There was a strong main effect of gender, with females exhibiting more positive condom attitudes than men (F = 10.3, df = 1, p<0.005).

### fMRI data

Data were thresholded at Z = 2.3 and cluster-corrected for multiple comparisons at p<0.05. Compared to the high MSV videos, low MSV videos were associated with greater response in the prefrontal cortex, including the inferior and middle frontal gyri, and the temporal cortex, including the bilateral middle temporal gyrus (Table 1a, Figure 2 [red-yellow activation]). The high MSV videos were associated with greater activity in occipital cortex, including the cuneus and the fusiform and lingual gyri, when contrasted with low MSV videos (Table 1b, Figure 2 [blue-green activation]). In addition, changes in the Condom Attitude Scale (ΔCAS) in favor of condom use were negatively correlated with brain activation in the bilateral Fusiform and Superior Temporal Gyri (Table 2). There were no significant differences in between African American (N = 11) and Caucasian (N = 16) participants response to either high or low MSV ads.

**Table 1:** 

Region^a^	Hem^f^	BA^b^	Size^c^	z-max^d^	X^e^	Y^e^	Z^e^
***a) Low MSV>High MSV***							
Middle Temporal Gyrus	L	21	3729	6.98	−54	−25	−4
Middle Temporal Gyrus	R	22	3149	6.56	44	−32	−2
Postcentral Gyrus	L	3	434	4.83	−50	−16	40
Lentiform Nucleus	L	N/A	433	3.84	−28	−4	−3
Medial Frontal Gyrus	L	6	431	4.49	−1	−3	59
Inferior Frontal Gyrus	L	47	414	4.08	−46	24	1
Insula	L	13	317	4.16	−45	−13	13
Precentral Gyrus	R	4	274	4.68	50	−13	39
***b) High MSV>Low MSV***							
Occipital Cortex	L	33	39498	6.88	−33	−70	−16
Middle Frontal Gyrus	R	6	933	4.25	29	−6	45
Caudate (head)	L	NA	932	4.1	−16	30	−2

a Location of the clusters and the local maxima of the BOLD fMRI signal change. *Z*>2.3 cluster corrected at *p*<0.05.

b Brodmann area.

c Number of voxels.

d *Z*-MAX values represent peak activation for the cluster.

e Talairach (1988) coordinates.

f Hemisphere.

*For clarity, clusters with less than 150 voxels are not reported in this table.*

**Table 2:** 

Region^a^	Hem^f^	BA^b^	Size^c^	z-max^d^	X^e^	Y^e^	Z^e^
**Lingual Gyrus**	R	18	833	4.05	25	−77	−9
**Superior Temporal Gyrus**	R	13	567	3.72	48	−42	15
**Fusiform Gyrus**	L	37	437	3.35	−41	−41	−14
**Superior Temporal Gyrus**	L	13	403	3.69	−41	−24	9

a Location of the clusters and the local maxima of BOLD fMRI signal change correlated with changes in Condom Use Attitude. *Z*>2.3 cluster corrected at *p*<0.05.

b Brodmann area.

c Number of voxels.

d *Z*-MAX values represent peak activation for the cluster.

e Talairach (1988) coordinates.

f Hemisphere.

### Primary PPI analyses

Averaging the z-scores (interaction with OCC seed) across all MTG/IFG voxels revealed that both conditions shared a negative overall PPI, with low MSV less negative than high MSV (−0.46 vs. −0.95). This finding was marginally significant (p = 0.060, two-tailed, n = 34).

The analysis by quintiles revealed an asymmetrical voxel distribution, as reflected by the increasing asymmetry between low MSV and high MSV from Quintile 1 to Quintile 5. That is, while the MTG/IFG voxels with the most negative z-score interactions with OCC during the low MSV condition did not differ from that during high MSV (Quintile 1: −2.83 vs. −2.86, p = 0.94), the most positive quintile of voxels was significantly greater during low MSV than high MSV (Quintile 5∶1.96 vs. 1.12, p = 0.0089). As seen in Table 3, proceeding from Quintile 1 to Quintile 5, a gradual divergence between low MSV and high MSV ads was observed, with low MSV interactions increasingly becoming more positive than their high MSV counterparts.

**Table 3:** 

Quintile	high MSVz-scorePPI voxel average	low MSV z-scorePPI voxel average	Difference	p-value two-tailedt-test (n = 34)	Standard Error(n = 34)
Quint 1 (Lowest 20%)	−2.86	−2.83	0.03	0.94	+/−0.20
Quint 2 (Lower 20%)	−1.74	−1.42	0.32	0.25	+/−0.19
Quint 3 (Middle 20%)	−1.01	−0.49	0.52	0.055	+/−0.20
Quint 4 (Higher 20%)	−0.24	0.47	0.71	0.012	+/−0.24
Quint 5 (Highest 20%)	1.12	1.96	0.84	0.0089	+/−0.33

*Psychophysiological Interaction (PPI) between regional masks derived from the High>Low MSV (Occipital Cortex, OCC) and High>Low Recognition Memory (Middle temporal and Inferior frontal gyri, MTG/IFG) under conditions of High MSV and Low MSV, averaged within quintiles of MTG/IFG voxels.*

## Discussion

This study extends findings from anti-smoking PSAs [17] to videos promoting safe sex through condom use. First, we confirmed our hypothesis that videos low in MSV would be better recognized both immediately and 3 weeks after the viewing session. This finding confirms the observation that high MSV video ads are not as well remembered as low MSV video ads [17] and suggests that the empirical strategy of capturing attention, i.e., “buying the eyeballs” [49], [50] may come at the expense of learning the content and therefore the message of the ad. In line with our hypotheses regarding the brain systems-level mechanism of this phenomenon, we found that video ads low in MSV activated the middle temporal gyrus (MTG), a brain region engaged in episodic memory encoding [18], [51] as well as the dorsal prefrontal cortex, a region associated with working memory and attention [19], [20], [21], [22] and considered to be a predictor of long-term message efficacy in several studies [35], [52], [53], [54]. By contrast, video ads high in MSV activated the occipital cortex (OCC), a region involved in primary processing of visual information [23], [24].

The symmetrically bilateral occipitoparietal activation associated with high MSV ads, extends findings first reported in Langleben et al. [17] and confirmed by Wang et al. [35] in studies of anti-tobacco ads. The occipital activation evoked by high MSV videos could reallocate processing resources away from encoding of ad messages [55], reflected in the lower MTG activation and reduced recognition memory for the high MSV compared to the low MSV ads. In addition, the negative correlation between increased CAS in favor of condom use and activation in the bilateral Fusiform and Superior Temporal Gyri also suggests that visual input could interfere with the cognitive processing of video ads’ message.

Finally, we used PPI to explore the hypothesis that relative to high MSV videos, low MSV videos allow for more efficient transfer of visual information inputs processed in the OCC and subsequently encoded in the MTG (also facilitated by co-activating attentional networks based in the IFG). By contrast, we hypothesized that high MSV videos should be associated with reduced interaction between these regions by reallocating brain processing resources away from the regions involved in processing and remembering ad messages [17], [56]. In support of the overstimulation hypothesis, we found that despite an overall negative level of interaction between these regions, the distribution of interactions was progressively more positive while viewing low MSV ads.

For the most part, the distribution of PPI z-scores was negative between the regions of interest during viewing of either high MSV or low MSV videos. Studies disagree to what extent the negative interactions between the occipital and rostral cortices are part of normal brain activity. There is some evidence that negative interactions may imply a dysfunctional inhibitory process. For example, relative to healthy control subjects, hyperactivation of the ventrolateral prefrontal cortex in patients with schizophrenia has been found to diminish top-down cognitive control normally mediated by the anterior cingulate cortex [57]. However, other fMRI studies consider negative interactions to be evidence of a more functional or efficient system. For example, a recent study, which examined the connectivity between the lateral and ventromedial prefrontal cortex, was characterized by primarily negative interactions, which the authors interpreted to be evidence of a complex dynamic system involving reciprocal interactions [58]. These findings are consistent with a hypothesis [59] that reduced negative interaction between cortical and limbic systems is a marker of major depression.

Despite the controversy about whether to interpret negative interactions as evidence of increased or decreased functional connectivity, our PPI analysis shows the negative interaction between the OCC and MTG/IFG regions to be weaker during the low MSV than high MSV at a marginally significant (p<0.06) level. This was especially prominent at the upper end of the PPI distribution (Table 3), suggesting that the former’s smaller mean (closer-to-zero) is a result of a shift towards more positive interactions during low MSV ads (rather than more negative interactions during high MSV). These findings provide support for the hypothesis that high MSV inhibits memory encoding as a result of increased activation of the primary sensory cortices, while low MSV videos may facilitate more efficient transfer of sensory information processed by OCC to downstream brain regions involved in encoding and attention (MTG, IFG). These results are consistent with theories modeling persuasion as a step-wise process that begins with attracting audience’s attention, followed by comprehension [25], where an excess of attention-grabbing devices could interfere with comprehension. They are also in-line with theories of dual-processing of information, such as the Elaboration Likelihood Model [6] and Limited Capacity theories, which postulate that sensory input could compete for the limited cognitive resources available to process various types of information in the message and interfere with subsequent processing that leads to successful encoding [60]. This conclusion also weighs on an earlier debate between the proponents and critics of the “distraction hypothesis” suggesting that distracting elements in an ad improve persuasiveness by reducing the resistance to persuasive messages [11], [61]. Our results complement the largely inconclusive behavioral studies of comparative effectiveness of the high and low MSV safe-sex video messages [3], [7], [62].

Although our results suggest that low MSV ads are more efficacious than the high MSV ads, they come with a number of caveats and limitations. Similarly to prior neuroimaging studies [37] [17], we used immediate and delayed recognition memory as a proxy outcome variable for the video ads’ behavioral impact. As the ultimate goal of neuroimaging research in the context of communications is to elucidate the mechanisms by which video ads effect sustained behavioral change and apply this knowledge towards improving video ad effectiveness, future research is necessary to confirm the link between remembering an ad and actually changing behavior in accordance with an ad’s message. The present study did not include in-depth standardized evaluation of sexual behaviors and participants’ emotional reaction of the video ads, both of which should be taken into account in future neuroimaging studies of safe-sex ad processing. While both ad and audience characteristics are important for the overall ad effect, the present study focused on ad characteristics, i.e. the MSV and accounted for the socio-demographic variables [63], [64], [65], [66], [67], [68] in the analysis. Future studies should investigate the influence of audience variables on ad effectiveness. Third, to preserve ecological validity, we used authentic previously aired ads of varying lengths, in their entirety. Though the high and low MSV categories did not differ on ad length, there remains a possibility that ad length variability could have confounded the Frame Recognition Test performance. Future research could consider alternative approaches, such as producing ads specifically for the purpose of research study or editing existing ads to standard size. Finally, since CAS is only a proxy outcome variable, longitudinal assessment of actual condom use following ad exposure would be required to determine the translational significance of our findings.

In conclusion, we confirmed prior reports [17] that high MSV video ads are more likely to interfere with and be less effective at conveying their messages than low MSV ads. Our neuroimaging findings suggest that the potential mechanism for this advantage may be that low MSV ads enable more efficient cross-brain communication between the brain regions involved in early processing of visual information and regions involved in downstream processing and encoding of this information into long-term memory. The fact that these behavioral and neuroimaging findings generalize across message genres justifies further efforts to incorporate neuroimaging in the evaluation and design of public health communications.
