# Correction: Low Message Sensation Health Promotion Videos Are Better Remembered and Activate Areas of the Brain Associated with Memory Encoding

**DOI:** 10.1371/journal.pone.0123143

**Published:** 2015-03-26

**Authors:** The PLOS ONE Staff

The third author's name is spelled incorrectly. The correct name is: Kanchana Jagannathan. The correct citation is: Seelig D, Wang A-L, Jagannathan K, Loughead JW, Blady SJ, Childress AR, et al. (2014) Low Message Sensation Health Promotion Videos Are Better Remembered and Activate Areas of the Brain Associated with Memory Encoding. PLoS ONE 9(11): e113256. doi:10.1371/journal.pone.0113256

There is an error in the last sentence of the “Behavioral data analysis” subsection of the Materials and Methods. The correct sentence is: A two-way repeated-measures ANOVA was performed comparing subject performance on Session 1 vs. Session 2 FRT (immediate vs. delayed sessions), and high MSV vs. low MSV. Independent t-tests were performed comparing gender performance.

There is an error in the first sentence of the “Behavioral Data” subsection of the Results. The correct sentence is: A two-way repeated-measures ANOVA revealed that memory performance was significantly better during the immediate post-task FRT session than the 3-week delayed session (F = 101.13, df = 1, p<0.001), and low MSV ads were bettered remembered than high MSV ads (F = 60.68, df = 1, p<0.001), but no interaction between Session and MSV (F = 2.20, df = 1, p = 0.15).

There is an error in the third to last sentence of the “Behavioral Data” subsection of the Results. The correct sentence is: Independent t-tests on gender revealed no significant differences between males vs. females in discrimination or response bias (Pr = 2.05 vs 2.20, t = -0.775, df = 32, p = 0.444; Br = 0.033 vs. 0.18, t = -1.67, df = 32, p = 0.104).

The last sentence of the “fMRI data” subsection of the Results, “There were no significant differences in between African American (N = 11) and Caucasian (N = 16) participants response to either high or low MSV ads,” should be deleted.

There is an error in the legend for Fig. 1. Please see the complete, corrected Fig. 1 here.

**Fig 1:**
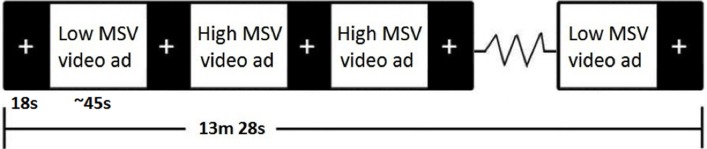
Design of the video task. The actual task displays 16 video messages (8 high MSV and 8 low MSV) in pseudorandom order (the order presented above is one possible organization).

There is an error in the legend for Fig. 2. Please see the complete, corrected Fig. 2 here.

**Fig 2:**
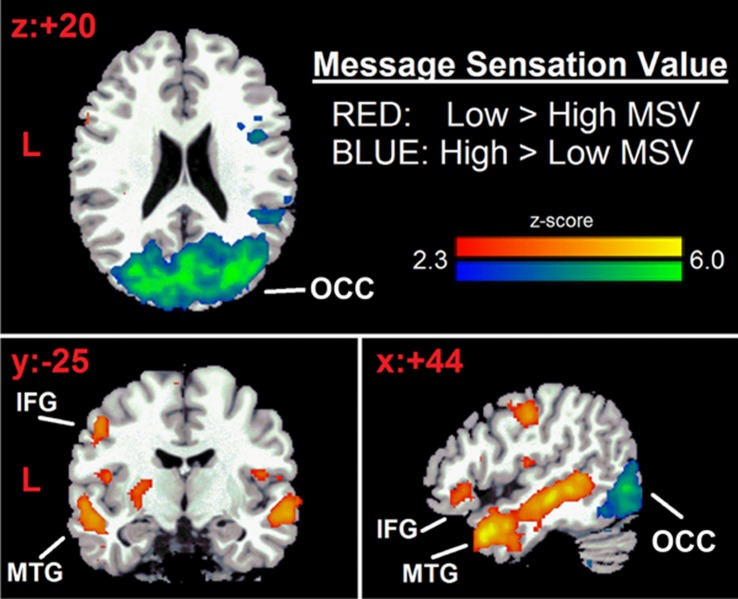
Brain response to safe-sex video messages. Middle temporal gyrus (MTG) and inferior frontal gyri (IFG) (red) have increased response for Low MSV>High MSV ads. Occipital cortex (OCC) (blue) has increased response for High MSV>Low MSV ads. Statistical maps are displayed over the Montreal Neurological Institute (MNI) brain template and thresholded at Z = 2.3, cluster-corrected for multiple comparisons at p<0.05. Coordinates converted to Talairach space [45].
